# Intolerance of Uncertainty and Cognition in Breast Cancer Survivors: The Mediating Role of Anxiety

**DOI:** 10.3390/cancers15123105

**Published:** 2023-06-08

**Authors:** Yesol Yang, Stephanie M. Gorka, Michael L. Pennell, Kellie Weinhold, Tonya Orchard

**Affiliations:** 1 Department of Internal Medicine, The Ohio State University Comprehensive Cancer Center-James, 406 W 10th Avenue, Columbus, OH 43210, USA; yan118@osumc.edu; 2 Department of Psychiatry and Behavioral Health, The Ohio State University Wexner Medical Center, 370 W 9th Avenue, Columbus, OH 43210, USA; stephanie.gorka@osumc.edu; 3 Institute for Behavioral Medicine Research, The Ohio State University, 460 Medical Center Drive, Columbus, OH 43210, USA; 4 Division of Biostatistics, College of Public Health, The Ohio State University, 1841 Neil Ave., Columbus, OH 43210, USA; pennell.28@osu.edu; 5 Human Nutrition Program, Department of Human Sciences, College of Education and Human Ecology, The Ohio State University, Columbus, OH 43210, USA; weinhold.8@osu.edu

**Keywords:** cognitive function, intolerance of uncertainty, breast cancer survivors, anxiety

## Abstract

**Simple Summary:**

Approximately 30% of breast cancer survivors experience cancer-related cognitive impairment (CRCI) after cancer treatments. Given that CRCI adversely affects quality of life and increases the risk of stroke and dementia, there is an urgent need to identify who might be more vulnerable to CRCI, and how to improve outcomes. Several studies have suggested the possibility that individuals who are sensitive to uncertainty (i.e., intolerance of uncertainty (IU)) are more likely to experience cognitive problems. Consistent with these studies, our findings also showed that greater IU is associated with higher anxiety, and such higher anxiety lowers perceived cognitive function. While much is still to be learned about this association, this study suggests that identifying those with IU and anxiety could assist with identifying those at higher risk for CRCI and that IU and anxiety may be potential targets for future intervention studies.

**Abstract:**

Cancer-related cognitive impairment (CRCI) is one of the most prevalent symptoms that breast cancer survivors experience. While cancer treatments are established contributors to CRCI, inter-individual differences in CRCI are not well understood. Individual differences in sensitivity to uncertainty are potential contributors to CRCI; however, no prior studies have attempted to examine this link in the context of breast cancer. To address the gap, we used preliminary findings from an ongoing cross-sectional study. A total of 38 women with stage I–III breast cancer (1–4 years post-treatment) were included in this study. Intolerance of uncertainty (IU) was assessed using the Intolerance of Uncertainty Scale. Self-reported cognitive function was assessed with the Neuro-QoL questionnaire. Anxiety was assessed using the Patient-Reported Outcomes Measurement System Bank. From this study, we found that anxiety mediates the association between IU and cognitive function of survivors. In other words, among post-menopausal breast cancer survivors, those with higher IU showed higher anxiety and consequently had lower cognitive function. This finding suggests that assessing IU may help predict the risk of CRCI. This study expands the current knowledge that addresses the importance of IU as a factor associated with cognitive health.

## 1. Introduction

Cancer-related cognitive impairment (CRCI) is one of the most prevalent symptoms that breast cancer survivors experience [1,2]. CRCI includes problems in memory, processing speeds, concentration, multitasking and word retrieval [3]. Some survivors report subtle or temporal CRCI, whereas others experience dramatic or permanent decline after treatment ends and long into cancer survivorship [4]. While cancer treatments are established contributors to CRCI, inter-individual differences in CRCI are not well understood [5]. Given that CRCI adversely affects quality of life and increases the risk of stroke and dementia [6,7], there is an urgent need to identify who might be more vulnerable to CRCI, and how to improve outcomes.

Individual differences in sensitivity to uncertainty are a potential risk factor for CRCI. Intolerance of uncertainty (IU) is defined as the trait-like tendency to appraise uncertain, ambiguous or uncontrollable situations as distressing or threatening [8,9,10,11,12]. Evidence has indicated that individuals with high IU interpret uncertain situations as threatening and display exacerbated negative effects and physiological arousal [13]. These maladaptive responses to uncertainty potentially contribute to the onset and maintenance of chronic anxiety [14]. As such, high IU has been conceptualized as a phenotypic core of internalizing disorders [15]. 

The possible mechanisms behind the IU–anxiety association were described by Grupe and Nitschke (2013), who introduced the Uncertainty and Anticipation Model of Anxiety (UAMA) theory of uncertainty and anxiety [16]. The UAMA model proposes neurobiological and psychological processes involved in adaptive responses under conditions of uncertainty [16,17,18]. This model indicates that anxiety occurs due to the increased expectancies about the probability and cost of unpredictable/uncertain future threats [16,17,18]. These biased expectancies result in individuals becoming hypervigilant and more attentive to possible threats, consequently developing clinical anxiety [16,17,18].

Although the exact underlying mechanism of the association between cognitive function and anxiety is unclear, several studies offer some potential clues. The attentional control theory contends that attention is regulated by two attentional systems (goal-directed and stimulus-driven), and anxiety modulates the balance between these systems [19,20]. In other words, increased anxiety breaks the balance between two attentional systems, and that broken balance negatively affects working memory and attention [20,21,22]. A recent meta-analysis also supports this theory by showing the strong relationship between anxiety and cognitive function, particularly in working memory [22]. Taken together, these findings show that anxiety can impact cognitive function.

Breast cancer survivors face uncertainty (e.g., fears about recurrence or long-term health concerns) [23,24,25] that has the potential to elicit anxiety about the future. A recent systematic review has found that 59% of survivors experience moderate fear about uncertainty, particularly cancer recurrence [26]. Experiencing uncertainty is common in breast cancer survivors throughout their illness trajectory, but each individual’s reactions toward uncertainty differ. Some survivors are intolerant to uncertainty (e.g., high IU) and exhibit increased levels of anxiety [23,24,25]. This suggests that among breast cancer survivors, those most sensitive to uncertainty may experience anxiety resulting in increased vulnerability to anxiety which can lead to cognitive deficits; however, to date, no prior studies have attempted to examine the role of IU on cognitive function in the context of breast cancer.

Therefore, to address the gap, we will investigate the association between IU and cognitive function among breast cancer survivors and the mediation effect of anxiety on this association. In this study, we hypothesized that higher IU would be associated with lower self-reported cognitive function among breast cancer survivors and that this association would be mediated by anxiety.

## 2. Materials and Methods

### 2.1. Design and Sample

This study presents preliminary findings from the ongoing cross-sectional study conducted at The Ohio State University (OSU) investigating associations of diet and other lifestyle factors, including psychosocial factors, with cognitive function in breast cancer survivors. A total of 38 female breast cancer survivors with complete data were enrolled in the study at the time of this analysis. Inclusion criteria for survivors were as follows:

- Female with a stage I–III breast cancer diagnosis;
- Between 1 and 4 years post-breast-cancer diagnosis;
- Post-menopausal (defined as at least 1-year post-menses);
- Aged 45–75 years;
- No diagnosis of diabetes;
- Ability to access and use internet resources, including video calls using the Zoom platform;
- Ability to read and understand English.

In this study, we limited inclusion to survivors 1–4 years post-treatment to reduce variability related to treatment trajectory. Previous studies have found that within 1-year-post-chemotherapy, breast cancer survivors exhibit the most varying degrees of CRCI compared with that seen immediately or more than 1 year after chemotherapy [27,28]. 

### 2.2. Recruitment and Enrollment

Potentially eligible women were identified through an electronic medical record review. Women were also recruited through electronic newsletters and by electronic and printed study fliers. Women were recruited between January 2022 and January 2023. Potentially eligible participants who expressed interest in participating in this study were invited to fill out a screening survey to determine study eligibility. After a screening survey was completed, the study team called the potentially eligible volunteer to describe the study in detail and confirm eligibility. If the individual was eligible and interested in participating in the study, informed consent and HIPAA authorization were obtained electronically and the participant was enrolled in the study. The study protocol was approved by The Ohio State University Cancer Institutional Review Board and all participants provided informed consent prior to study entry. The study was listed at ClinicalTrials.gov with identifier NCT05048108.

### 2.3. Data Collection

This was a fully remote study. Data were collected from breast cancer survivors online within 1–2 weeks of obtaining written informed consent. Questionnaires for data collection were built into REDCap (Research Electronic Data Capture), a secure, web-based, HIPAA-compliant data collection platform. The questionnaires covered demographics, medical history, breast cancer history, anxiety, cognitive function, and intolerance of uncertainty. 

Intolerance of uncertainty was assessed using the Intolerance of Uncertainty Scale (IUS-12) [10]. Items on the IUS-12 are rated on a five-point Likert-type scale, with 1 indicating that the statement is not at all characteristic of the respondent, and 5 indicating that the statement is entirely characteristic of the respondent. The IUS-12 produces a total score ranging from 12 to 60, with higher scores indicating greater IU. In the present sample, an internal reliability of IUS-12 total was good (α = 0.89).

Self-reported anxiety was assessed using the Patient-Reported Outcomes Measurement System (PROMIS) Bank v1.0 (computer adaptive test (CAT)) [29]. With a CAT, the participants’ responses guided the system’s choice of subsequent items from the full item bank. Although the items differed across respondents taking a CAT, the scores were comparable across participants. Participants were asked to respond to questions about the severity of anxiety over the past 7 days on a five-point Likert type scale. These questions were a subset of items from a larger item bank that demonstrated high content validity and reliability. Item scores were summed to obtain the total raw score which was then converted to a T-score (mean 50, SD = 10) and rounded to the nearest integer for ease of reporting [30]. Higher scores for anxiety reflect higher levels of anxiety.

Self-reported cognitive function was assessed with the validated 8-item Neuro-QoL v2.0 questionnaire (short-form) [31]. Each item was rated on a Likert scale of 1–5 (from 1 = very often to 5 = never). Raw scores ranged from 8 to 40 and standardized T-scores were generated with a mean of 50 and standard deviation of 10. Higher T-scores indicate better cognitive function. In the present sample, the internal reliability of Neuro-QoL total was excellent (α = 0.92).

### 2.4. Data Analysis

Three analyses were conducted using SPSS 28.0 (SPSS Inc., Chicago, IL, USA). First, descriptive statistics were calculated to summarize survivors’ demographic and clinical information. Second, the Kolmogorov–Smirnov test showed that only anxiety and self-reported cognitive function fit the normal distribution. Thus, to assess bivariate relationships between IU, anxiety, and self-reported cognitive function, nonparametric Spearman’s correlations were used. Third, a mediation analysis was performed taking IU as a predictor (X), anxiety as a mediator (M), and self-reported cognitive function as the outcome variable (Y). We confirmed that error terms were normally distributed. Model 4 of the PROCESS plug-in SPSS [32] was used to test the indirect effect of IU on self-reported cognitive function via anxiety. The direct and indirect effects were estimated using a percentile bootstrap estimation approach with 5000 samples (95% bias-corrected confidence intervals) [33], implemented with the PROCESS macro version 4.2 beta [34,35]. This model included one direct effect (the effect of IU on self-reported cognitive function) and one indirect effect (the effect of IU on self-reported cognitive function via anxiety), both adjusted for age.

## 3. Results

### 3.1. Sample Characteristics

The thirty-eight breast cancer survivors in this study had a mean (SD) age of 58.6 (SD = 8.7) years. Among the thirty-eight female breast cancer survivors in this study, the majority were White (92%), married or cohabiting with a partner (74%), and had at least a bachelor’s degree (79%). A total of 45% of study participants reported that they worked full-time. The majority of survivors who participated in this study had been diagnosed with stage I breast cancer (53%), had surgeries for breast cancer removal (100%), and had been treated with anti-hormone therapy (71%). The means IU, anxiety, and self-reported cognitive function were 24.5 (SD = 7.9), 51.5 (SD = 7.2), and 48.1 (SD = 8.5), respectively. Detailed demographic and clinical information of survivors is summarized in Table 1.

### 3.2. Correlation Analysis between IU, Anxiety, and Self-Reported Cognitive Function 

**Figure 1:**
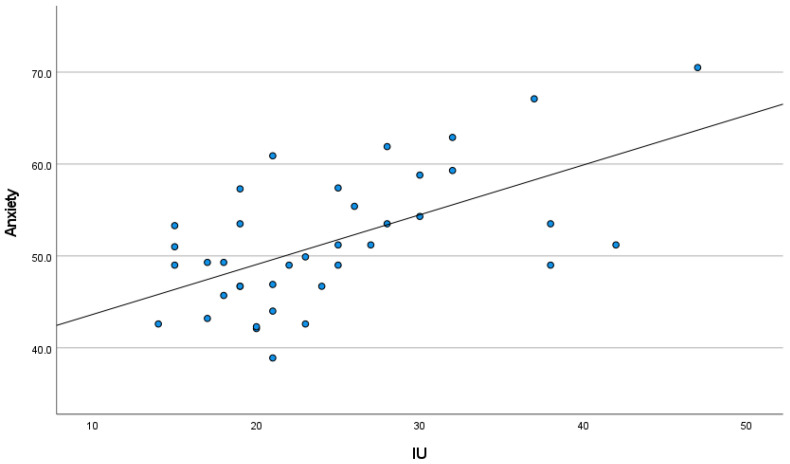
Association between IU and anxiety, with linear trends superimposed.

**Figure 2:**
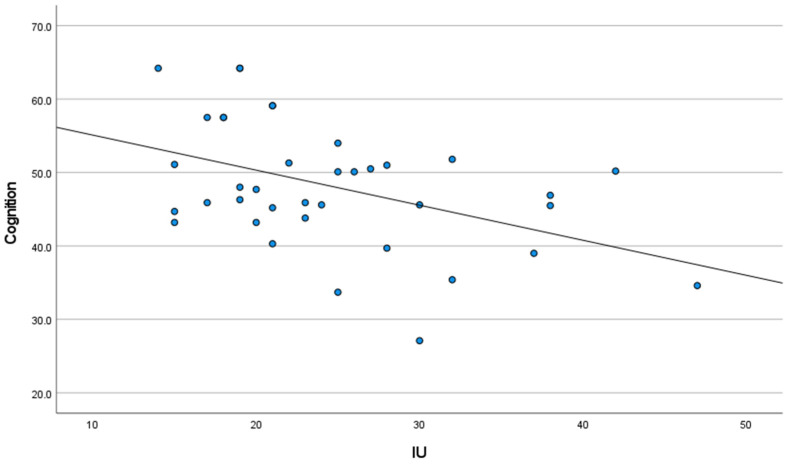
Association between IU and self-reported cognition, with linear trends superimposed.

**Figure 3:**
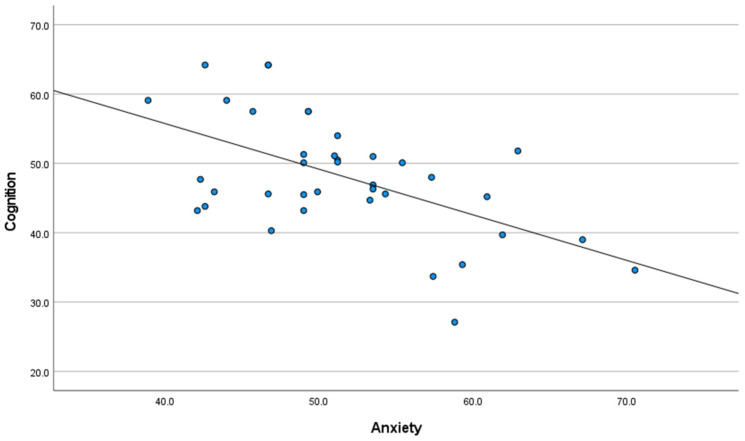
Association between self-reported cognition and anxiety, with linear trends superimposed.

As noted in Figure 1, IU was significantly associated with anxiety (r = 0.55, 95% CI [0.28, 0.74], *p* = 0.01), and Figure 2 presents that IU was significantly associated with self-reported cognitive function (r = −0.39, 95% CI [−0.63, −0.08], *p* = 0.05). Additionally, Figure 3 presents that a significant negative association was found between anxiety and self-reported cognitive function (r = −0.44, 95% CI [−0.67, −0.14], *p* = 0.01).

### 3.3. The Mediation Effect of Anxiety on the Association between IU and Self-Reported Cognitive Function

**Figure 4:**
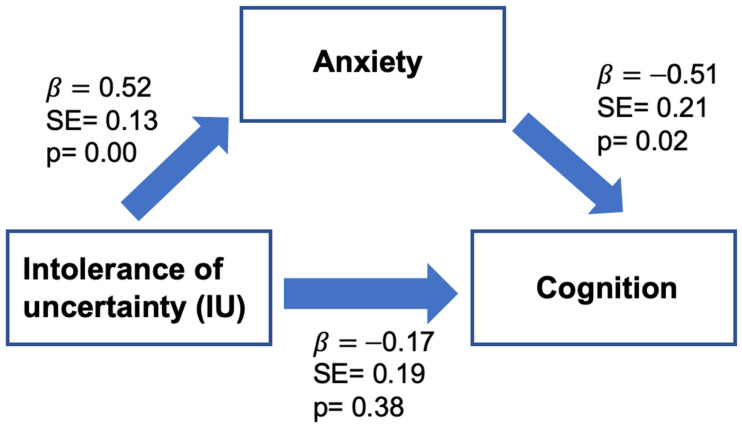
Model of the mediation effect of anxiety on the association between IU and cognitive function of breast cancer survivors.

Regression analysis was used to investigate the hypothesis that anxiety mediates the effect of IU on self-reported cognitive function, after controlling for age (Figure 4). The indirect effect was significant: β = −0.26, SE = 0.12, 95% CI [−0.54, −0.06]. This result can be interpreted to mean that for every 1-point increase in IU, there was a 0.26-point decrease in self-reported cognitive function that was mediated by anxiety. The direct effect of IU on self-reported cognitive function was 35% smaller than the direct effect and was insignificant (β = −0.17, *p* = 0.38). Approximately 34% of the variance in self-reported cognitive function was accounted for by anxiety, IU, and age (R^2^ = 0.34). 

## 4. Discussion

Consistent with our hypothesis, anxiety mediates the association between IU and the self-reported cognitive function of breast cancer survivors. In other words, greater IU is associated with higher anxiety, and such higher anxiety lowers self-reported cognitive function. This result shows that the effect of IU on self-reported cognitive function exists when anxiety mediates its relationship. 

Our findings on the association between IU and anxiety are explained by research illustrating neural mechanisms associated with IU and anxiety. Grupe and Nitschke (2013) showed evidence that anxious pathology is linked to the frontolimbic neural circuit, and that this circuit is also involved in responding to uncertainty [16]. Within this circuit, the anterior insula (aINS) is thought to be a core node that integrates information about internal and external stimuli to produce interoceptive awareness, and generates anticipatory emotional responses for future events [36,37]. Consistent with these findings, several non-cancer studies have reported that those with greater IU show aINS hyperactivation, and those with hyper aINS activation tend to report higher anxiety [38,39,40,41]. These findings suggest that it is possible that breast cancer survivors with higher IU may experience higher anxiety when they face uncertainty, and that these links can be altered by aINS functioning. Therefore, future studies that investigate the links between IU, anxiety, and aINS functioning among breast cancer survivors are needed. This understanding gained will ultimately contribute to developing interventions with precise brain function targets to treat anxiety.

Our results also revealed that anxiety is linked to self-reported cognitive function. One possible explanation for this relationship is that anxious individuals have alterations in aINS functioning and that alterations can negatively affect cognitive function [42,43]. Several studies have reported that aINS plays a role in facilitating information processing by initiating appropriate signals to engage brain areas mediating attention and executive function [42,43]. Similarly, a recent review has found that anxiety is a contributing factor for CRCI, although the mechanisms underlying this association are not well stated [44]. Thus, more neuroimaging research is needed to elucidate the brain risk phenotype of cognitive problems in breast cancer survivors. Furthermore, more specific cognitive domains (e.g., attention, memory, executive function) should be included in such research to develop more specific prevention and treatment strategies for those with cognitive problems. 

In this study, we used a self-report instrument that asks participants to rate their subjective tolerance of uncertainty [9]; however, there are several other ways to assess IU. One way is to use neuroimaging methods (e.g., fMRI) that expose participants to uncertain stressors (U-Threat) and measure their neural reactivity [45,46]. U-Threat is defined as uncertainty about a possible future threat in its timing, intensity, and/or duration [45,46]. It relates to potentially harmful situations, but not to an immediate threat [45,46]. Several studies suggest that the degree of aINS activation to unpredictable or uncertain threats (i.e., U-Threat) reflect individual differences in sensitivity to uncertainty [45,46,47,48], and that this is a more objective and specific index for IU than that of self-report measures [40,49,50]. Thus, further studies of breast cancer survivors should include both subjective and objective IU indices to validate the relationships between IU, anxiety, and cognitive function. An enhanced understanding of this association will facilitate the identification of breast cancer survivors at risk of CRCI and help develop interventions for those with CRCI.

Together, this study suggests the importance of IU and anxiety on cognitive function among female breast cancer survivors following treatment completion, therefore highlighting potential targets for intervention. Furthermore, psychological interventions that help breast cancer survivors relieve anxiety and become more tolerant of uncertainty may facilitate cognitive recovery. In clinical settings, cognitive behavioral therapy, which has been shown to be effective for anxiety disorders, can be used to treat CRCI [51]. Through this therapy, cancer survivors may alter their maladaptive emotional responses by changing their thoughts, behaviors, or both [51]. Additionally, Intolerance of Uncertainty therapy, developed to treat generalized anxiety disorder, can be used for cancer survivors with cognitive problems. This therapy could help survivors reevaluate uncertainty as a normal part of their lives and empower them to make decisions and participate in everyday activities despite this uncertainty [52,53]. In addition to the IU therapy, healthcare providers may encourage breast cancer survivors to get involved in activities that promote supportive social relationships. Doing so may offset the risk of CRCI through regulating aINS activation. The literature shows that individuals who receive greater assistance from others (i.e., higher social support) better manage uncertainty, leading to a decrease in anxiety [54]. In contrast, without social support, those with high IU remain chronically anxious, resulting in negative cognitive outcomes [55,56]. A possible explanation for this finding is that social support reduces brain activation within aINS, which in turn buffers the impact of stressors on health [57]. Without social support, aINS will remain activated, consequently leading to brain circuit dysfunction [57]. More research is needed to determine the effectiveness of interventions that reduce anxiety and IU and consequences on CRCI in cancer survivors.

This study has strengths which include a post-menopausal breast cancer cohort with extensive demographic, clinical, psychological, and cognitive data. However, this study has several limitations. First, this study was cross-sectional, and we were unable to establish the temporality between IU, anxiety, and self-reported cognitive function. Although the model we diagrammed is the most clinically plausible mechanism, future studies with repeated measures over time would provide a more comprehensive understanding of the associations among those variables. Second, the measure of cognitive function used in this study was specific to the subjective perception of an individual’s overall cognitive function. Future studies should include each specific aspect of the cognitive domain (e.g., memory or attention) to better understand which specific cognitive function is associated with IU and anxiety. Additionally, future studies should include other potential confounders of this relationship such as fatigue and/or lifestyle factors (exercise, diet) that were not included in this analysis, but may contribute to anxiety, intolerance of uncertainty, and cognitive function. Lastly, the study participants were predominantly Non-Hispanic White (92%). In addition, we only included breast cancer survivors 1–4 years post-treatment. This could decrease the generalizability of the study results. Future studies should include participants from diverse ethnic and racial groups and treatment trajectories.

## 5. Conclusions

In summary, we find that post-menopausal breast cancer survivors with higher IU showed higher anxiety and consequently reported lower cognitive function than those survivors with lower IU. This finding suggests that identifying those with higher IU and screening post-menopausal women with breast cancer for high IU could be a strategy to identify those at higher risk for CRCI. This study will contribute to the current knowledge that shows the importance of IU as a factor associated with cognitive health.

## Figures and Tables

**Table 1:** Sample characteristics (n = 38).

	n	%
Race/Ethnicity		
Non-Hispanic White	35	92
Black or African American	3	8
Education		
Associate degree	5	13
Some college course work completed	3	8
Bachelor’s degree	14	37
Advanced degree (master, doctorate, medical, etc.)	16	42
Marital status		
Single (unmarried, divorced, widowed, etc.)	10	26
Married or cohabiting with partner	28	74
Employment status		
Work 40+ hours a week	17	45
Work fewer than 40 h a week	9	24
Homemaker	1	2
Retired	11	29
Cancer stage		
I	20	53
II	9	24
III	8	21
Unknown/I do not know	1	2
Cancer treatment (select all that apply)		
Surgery—lumpectomy/partial mastectomy	18	47
Surgery—total (simple) mastectomy	13	34
Surgery—modified radical mastectomy	7	18
Radiation	25	66
Chemotherapy	22	58
Anti-hormone therapy	27	71
Targeted therapy	7	18
Immunotherapy	1	3

## Data Availability

The data will be accessible upon reasonable request to the corresponding author.
